# Supplementary material for: Elevated Plasma Levels of Circulating Extracellular miR-320a-3p in Patients with Paroxysmal Atrial Fibrillation
Source: Int J Mol Sci. 2020 May 15;21(10):3485. doi: 10.3390/ijms21103485 (PMC7279020; doi:10.3390/ijms21103485)

## Supplementary material

**Table S1.** Mann–Whitney (with Bonferroni–Holm correction) p-values of significance for comparisons of the study sample characteristics between the study sample groups: PAF: 30 paroxysmal atrial fibrillation patients; HT: 30 hypertensive patients without atrial fibrillation; CONTR: 30 healthy controls. LDL: low density lipoproteins; HDL: high density lipoproteins; A414 and A385: spectrophotometric absorbance at 414 and 385 nm wavelengths obtained during hemolysis assessment, respectively; HS: hemolysis score; dCq (miR-23a-3p–miR-451a): Cq difference between miR-23a-3p and miR-451a.

| Sample characteristics | Group comparison | P-value |
|---|---|---|
| | CONTR vs. HT | 0.000183070000 |
| Age | PAF vs. CONTR | 0.000000017523 |
| | PAF vs. HT | 0.002294048000 |
| | CONTR vs. HT | 0.013398570000 |
| Total cholesterol, mmol/L | PAF vs. CONTR | 0.212813500000 |
| | PAF vs. HT | 0.189015500000 |
| | CONTR vs. HT | 0.009924119000 |
| Triglycerides, mmol/L | PAF vs. CONTR | 0.074259770000 |
| | PAF vs. HT | 0.337305400000 |
| | CONTR vs. HT | 0.004906899000 |
| LDL cholesterol, mmol/L | PAF vs. CONTR | 0.189015500000 |
| | PAF vs. HT | 0.167135200000 |
| | CONTR vs. HT | 0.289455400000 |
| HDL cholesterol, mmol/L | PAF vs. CONTR | 0.095566180000 |
| | PAF vs. HT | 0.078953960000 |
| | CONTR vs. HT | 0.066157980000 |
| Left atrial volume, mL | PAF vs. CONTR | 0.000000260383 |
| | PAF vs. HT | 0.000423750500 |
| | CONTR vs. HT | 0.005143373000 |
| A414 | PAF vs. CONTR | 0.115067700000 |
| | PAF vs. HT | 0.167135200000 |
| | CONTR vs. HT | 0.248849900000 |
| Δ(A414-A385) | PAF vs. CONTR | 0.337305400000 |
| | PAF vs. HT | 0.424012800000 |
| | CONTR vs. HT | 0.157975700000 |
| HS | PAF vs. CONTR | 0.248849900000 |
| | PAF vs. HT | 0.356556200000 |
| | CONTR vs. HT | 0.199523400000 |
| A414/A385 Ratio | PAF vs. CONTR | 0.229179000000 |
| | PAF vs. HT | 0.356556200000 |
| | CONTR vs. HT | 0.089782710000 |
| dCq (miR-23a-3p–miR-451a) | PAF vs. CONTR | 0.248751400000 |
| | PAF vs. HT | 0.255656700000 |

**Table S2.** Characteristics of qPCR detection for analyzed miRNAs in two samples with three technical replicates. Prior to cDNA synthesis, miRNA samples were spiked-in with synthetic miRNA cel-miR-39-3p in final concentrations of 1 and 10 pM in Sample_01 and Sample_02, respectively. Cq: qPCR quantification cycle; SD: standard deviation; CV: coefficient of variance between three qPCR technical replicates.

We observed the difference of more than 10 cycles in Cq values of cel-miR-39-3p between 10 and 1 pM spike-in miRNA added to the sample. Low Cq value of cel-miR-39-3p was possibly the result of degradation of synthetic miRNA during cDNA synthesis. Thus, spike-in control should be added to miRNA sample in the final concentration of not less than 10 pM, which is equivalent to 1.2 × $10^7$ copies of cel-miR-39-3p per one cDNA synthesis reaction.

| miRNA | Sample_01 | | | Sample_02 | | |
|---|---|---|---|---|---|---|
| | **Mean Cq (SD)** | **Mean relative expression level × $10^4$ (SD)** | **CV, %** | **Cq mean (SD)** | **Mean relative expression level × $10^4$ (SD)** | **CV, %** |
| hsa-miR-16-5p | 14.16 (0.12) | - | - | 14.87 (0.15) | - | - |
| hsa-miR-146a-5p | 21.65 (0.10) | 55.98 (3.21) | 11.0 | 23.28 (0.03) | 29.43 (1.89) | 7.7 |
| hsa-miR-150-5p | 20.43 (0.08) | 130.27 (3.37) | 4.9 | 21.63 (0.06) | 92.47 (2.96) | 5.2 |
| hsa-miR-19a-3p | 20.56 (0.04) | 118.47 (5.26) | 6.7 | 21.73 (0.16) | 85.95 (1.62) | 4.4 |
| hsa-miR-21-5p | 18.67 (0.05) | 441.91 (16.55) | 8.5 | 20.19 (0.12) | 251.69 (13.74) | 8.7 |
| hsa-miR-23a-3p | 23.02 (0.21) | 21.65 (1.63) | 8.9 | 24.54 (0.11) | 12.37 (0.53) | 9.8 |
| hsa-miR-29b-3p | 21.80 (0.07) | 50.33 (0.99) | 3.0 | 22.88 (0.15) | 38.62 (0.01) | 2.8 |
| hsa-miR-320a-3p | 20.91 (0.13) | 93.39 (3.18) | 7.8 | 22.53 (0.14) | 49.36 (0.15) | 0.5 |
| hsa-miR-328-3p | 26.68 (0.11) | 1.73 (0.09) | 11.7 | 28.69 (0.08) | 0.7 (0.04) | 10.3 |
| hsa-miR-375-3p | 24.36 (0.02) | 8.51 (0.2) | 5.2 | 26.55 (0.12) | 3.05 (0.06) | 3.7 |
| hsa-miR-409-3p | 31.00 (0.23) | 0.09 (0.01) | 7.4 | 29.83 (0.10) | 0.32 (0.03) | 11.3 |
| hsa-miR-451a | 12.31 (0.11) | 36265.41 (969.37) | 6.2 | 12.54 (0.25) | 46331.6 (1617.94) | 5.1 |
| cel-miR-39-3p | 41.33 (1.74) | - | - | 30.95 (0.16) | - | - |

**Table S3.** P-values of significance of relationship between medical therapies potentially influencing platelet state and miRNA relative plasma levels obtained by MLR analysis in a combined PAF+HT group (N=60) with age and hemolysis indices as a confounding factors. ACEI: angiotensin-converting enzyme inhibitors; HS: hemolysis score; dCq (miR-23a-3p–miR-451a): Cq difference between miR-23a-3p and miR-451a.

| miRNA | miR-146a-5p | miR-150-5p | miR-19a-3p | miR-21-5p | miR-29b-3p | miR-320a-3p | miR-328-3p | miR-375-3p | miR-409-3p |
|---|---|---|---|---|---|---|---|---|---|
| Beta-blockers | 0.941 | 0.352 | 0.352 | 0.941 | 0.352 | 0.941 | 0.714 | 0.352 | 0.941 |
| Calcium channel blockers | 0.954 | 0.802 | 0.954 | 0.990 | 0.954 | 0.954 | 0.184 | 0.990 | 0.954 |
| ACEI | 0.017 | 0.834 | 0.771 | 0.017 | 0.151 | 0.050 | 0.635 | 0.635 | 0.771 |
| Antiplatelet drugs | 0.852 | 0.703 | 0.783 | 0.211 | 0.211 | 0.211 | 0.860 | 0.703 | 0.211 |
| Anticoagulants | 0.623 | 0.117 | 0.726 | 0.329 | 0.574 | 0.623 | 0.574 | 0.726 | 0.726 |
| Statins | 0.770 | 0.489 | 0.495 | 0.759 | 0.770 | 0.759 | 0.495 | 0.770 | 0.489 |
| Age | 0.546 | 0.001 | 0.546 | 0.633 | 0.546 | 0.692 | 0.546 | 0.692 | 0.510 |
| HS | 0.598 | 0.058 | 0.169 | 0.367 | 0.289 | 0.289 | 0.470 | 0.289 | 0.524 |
| dCq (miR-23a-3p –miR-451a) | 0.068 | 0.001 | 0.001 | 0.128 | 0.044 | 0.552 | 0.257 | 0.000 | 0.260 |

**Figure S1.** Distribution plots of the study sample characteristics in the study sample groups: PAF: 30 paroxysmal atrial fibrillation patients; HT: 30 hypertensive patients without atrial fibrillation; CONTR: 30 healthy controls. The boxplots represent median and interquartile ranges (IQR) in the box, minimum and maximum values in the "whiskers", and outliers in the rhombic dots. LDL: low density lipoproteins; HDL: high density lipoproteins; A414 and A385: spectrophotometric absorbance at 414 and 385 nm wavelengths obtained during hemolysis assessment, respectively; HS: hemolysis score; dCq (miR-23a-3p–miR-451a): Cq difference between miR-23a-3p and miR-451a.

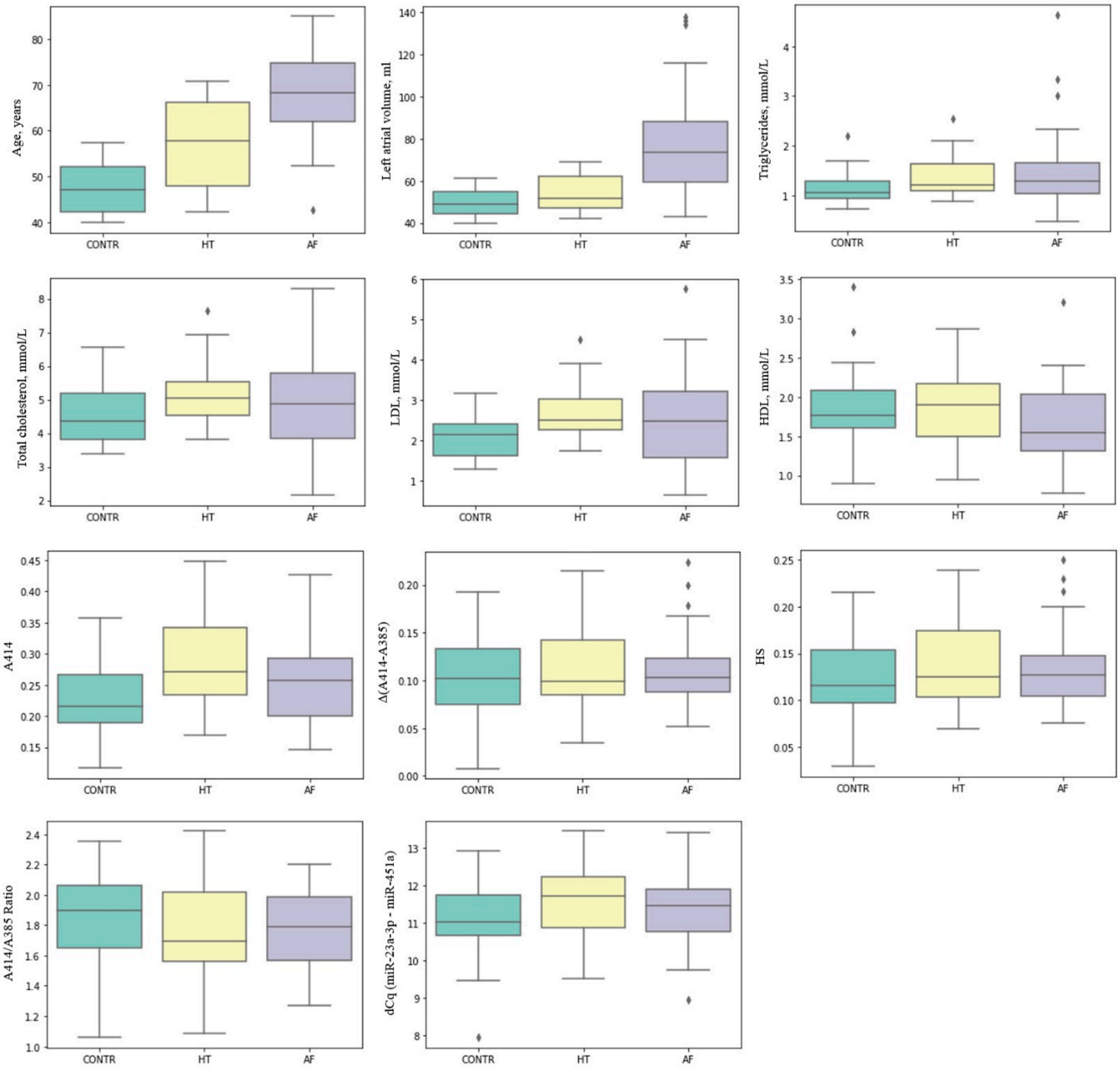

Supplement: Supplementary file 1 [file ijms-21-03485-s001.pdf]
